# Synergistic inhibition of MEK/ERK and BRAF V600E with PD98059 and PLX4032 induces sodium/iodide symporter (NIS) expression and radioiodine uptake in BRAF mutated papillary thyroid cancer cells

**DOI:** 10.1186/s13044-018-0057-6

**Published:** 2018-10-11

**Authors:** Honglai Zhang, Dong Chen

**Affiliations:** 1 Department of Thyroid Surgery, the affiliated Hospital of Qingdao University, Qingdao, Shandong China; 2 Department of General Surgery, the affiliated Hospital of Qingdao University, Qingdao, Shandong China

**Keywords:** Papillary thyroid carcinoma, Assodium/iodide symporter, Mutation BRAF^V600E^, Mitogen-activated protein kinase

## Abstract

**Background and Aims:**

The activating mutation BRAF^V600E^ is a frequent genetic event in papillary thyroid carcinomas (PTC). Mutation BRAF^V600E^ is associated with the loss of a sodium/iodine symporter (NIS), and subsequent radioiodide-refractory (RAI) metastatic disease. Use of BRAF V600E inhibitors could partly restore NIS expression and Iodide uptake by inhibition of mitogen-activated protein kinase (MAPK) pathway. Previous study has reported that the BRAF V600E inhibitors could re-activate MAPK signals. In the present study, we investigated whether the combination treatment of BRAF V600E inhibitor and MAPK signal inhibitor could more effectively increase NIS expression and RAI uptake, and explore the mechanisms.

**Methods:**

BCPAP and K1 cells were exposed to increasing concentrations of BRAF V600E inhibitor PLX4032 (0.01 μM, 0.1 μM, 1 μM) or MEK/ERK inhibitor PD98059 (0.01 μM, 0.1 μM, 1 μM) or with their association or/and in the presence of 3 mM perchlorate (ClO^−^ _4_) for 0–72 h. Iodide uptake and expression of BRAF, phosphorylated (p) ERK1/2, NIS were detected.

**Results:**

PLX4032 or PD98059 alone did not induce NIS expression and increase Iodide uptake in BCPAP and K1 cells. But combined treatment of PLX4032 and PD98059 significantly induce NIS expression and increase Iodide uptake in BCPAP and K1 cells. PLX4032 alone inhibited p-ERK expression at early time, and re-activated p-ERK expression at late time. However, combined treatment of PLX4032 and PD98059 completely inhibited p-ERK expression.

**Conclusion:**

Simultaneously suppressing BRAF V600E and p-ERK restored NIS expression and increase Iodide uptake in PTC cells, which was associated the inhibition of p-ERK expression. The results warrants clinical trials to confirm.

## Background

Differentiated thyroid cancer follicular cells have the ability to absorb iodine, which is the theoretical basis for radioactive iodine (RAI) treatment of differentiated thyroid cancer [1]. RAI is the standard treatment for differentiated thyroid cancer at present. If patients with RAI-ineffective thyroid cancer have lost surgical indications, any other method currently used clinicall cannot be curing. This is the main cause of thyroid cancer-related morbidity and mortality. Sodium iodide cotransporter (NIS) mediates the active iodide uptake at the basolateral membrane of the thyroid follicular cell, plays a crucial role in the success of radioiodine therapy [2]. Thyroid-stimulating hormone receptor (TSHR) up-regulates NIS and related molecular processes [2]. The main mechanism of RAI-refractory thyroid cancer is the abnormal silencing of NIS and TSHR gene in the thyroid cancer cells [2]. NIS is negatively regulated by the MAPK pathway in thyroid cancer, in which the BRAF V600E mutation plays an important role [3].

BRAF600E (valine-to-glutamate, position 600) is the second most common mutation in human cancer, and the most common oncogenic mutation in thyroid cancer [4]. Mutated BRAF600E protein results in increased mitogen-activated protein kinase (MAPK) activity (MEK1/2/ERK1/2) [5]. It has previously reported that targeting MAPK pathway using MEK inhibitors could increased thyroid gene expression and radioiodine uptake in thyroid cancer cells [6, 7] and melanoma [8]. We therefore suggested that targeting BRAF/MAPK or MEK/ERK pathway could be clinically effective in restoring RAI avidity in RAI-refractory thyroid cancer.

It has been recently found that using dabrafenib, the BRAF V600E inhibitor could partially induce radioiodine uptake in RAI-refractory thyroid cancer in patients [9], suggesting that the therapeutic effectiveness of RAI treatment using single agents was limited. PLX4032 (vemurafenib) is a selective small molecule inhibitor of BRAF V600E, showing a preferential inhibition of BRAF V600E-positive metastatic melanoma [10-12]. Data from preliminary studies suggest that PLX4032 might have activity in BRAFV600E-positive papillary thyroid cancer [13, 14]. Brose et al. has recently reported that Vemurafenib showed antitumour activity in patients with progressive, BRAFV600E-positive papillary thyroid cancer refractory to radioactive iodine who had never been treated with a multikinase inhibitor [15]. The mutation of the BRAFV600E gene is related with greater resistance to postoperative treatment with 131I since the onset of the disease [16]. A new study suggests that the PLX4032 may be effective in the 25 to 50% of patients with papillary thyroid cancer who develop resistance to radioactive iodine, a standard treatment. This image illustrates a multimodal therapeutic strategy for an iodine-refractory BRAF-mutated metastatic papillary thyroid carcinoma with reversed radioiodine resistance using BRAF inhibitors. As such, this agent represents a potential new treatment option for these patients.

We have recently found that treatment of thyroid cancer cells with PLX4032 alone resulted in a transient inhibition of pERK1/2 expression at 4–6 h, but quickly lead to paradoxically increased ERK signalingat 24–48 h, which maked the thyroid cancer cells resistant to PLX4032 treatment [17]. Therefore, we hypothesize that simultaneously suppressing BRAF V600E and ERK1/2 signaling could suppress the re-activation of ERK1/2 signaling by PLX4032, which may likely have a synergistic effect on thyroid gene expression and RAI uptake in thyroid cancer cells with BRAF V600E mutation.

In our study, we tested the effect of BRAF V600E inhibitor PLX4032 or in combination of the MEK/ERK inhibitor PD98059 on RAI uptake and NIS expression, in papillary thyroid cancer cell (PTC) with mutated BRAF600E.

## Methods

### Cell cultures

BRAF^V600E^-positive papillary thyroid cancer cell BCPAP and K1 cells were obtained from the German Collection of Microorganisms and Cell Culture (DSMZ, Braunschweig, Germany). They were authenticated by short tandem repeat profiling, as previously described [18]. Cells were grown in humidified incubator at 37 °C with 5% CO_2_, 6H hormone (1 mU/ml bovine TSH, 10 μg/ml bovine insulin, 10 nM hydrocortisone, 5 μg/ml transferrin, 10 ng/ml somatostatin, and 2 ng/ml l-glycyl-histidyllysine) and 10% fetal bovine serum in RPMI 1640 medium. Cells were expanded to 70–80% confluence and then used for the experiments described below.

### Western blot

BCPAP and K1 cells (1.5 × 10^5^ cells per well) were seeded in 96-well cell culture plates and harvested at 70 to 80% confluence. Then the cells were exposed to increasing concentrations of the BRAF V600E inhibitor PLX4032 (0.01 μM, 0.1 μM, 1 μM) or the ERK inhibitor, PD98059 (0.01 μM, 0.1 μM, 1 μM) or with their association for 2, 6, 12, 24, 48, 72 h. Cell lysates were prepared in 0.5% Triton X-100, 150 mM NaCl, 5 mM Tris supplemented with 1 × Halt protease inhibitor cocktail and 1 × Halt phosphatase inhibitor cocktail (Pierce, Rockford, IL), centrifuged at 3500 r.p.m. for 10 min at 4 °C. Protein concentration was measured using a Bio-Rad protein assay (Bio-Rad Laboratories, Hercules, CA). Proteins resolved by SDS–PAGE were electrophoretically transferred to polyvinylidene difluoride membrane and Western blots were carried out using standard techniques. The primary antibodies were as below: phosphorylated (p) ERK1/2 and ERK antibody (Santa Cruz Biotechnology, Shanghai, China); BRAF (sc-5284) (Santa Cruz Biotechnology); NIS (Protein tech). Anti-β-actin (Santa Cruz Biotechnology).

Primary antibody was incubated overnight at 4 °C in 5% milk/PBST. Membranes were washed with PBST and incubated with HRP-conjugated secondary antibodies diluted in 5% milk/PBST. Bands were visualised with enhanced chemiluminescence developed with X-ray film. Densitometry was performed (Kodak MI SE software, Carestream Health), and protein quantities recorded relative to anti-β-actin. All samples were analysed at least in triplicate.

### Iodide uptake assays

Iodide uptake was measured as previously described [19]. Briefly, cells (1.5 × 10^5^) were seeded in 6-well plates and then incubated with PLX4032 or PD98059 alone or their combination at 0.1 μM for 24–72 h. Then the cells were incubated with 2 μCi NaI125 in 5 μM nonradioactive NaI for 30 min at 37 °C with 5% CO2. Cells were then washed with cold Hank’s Balanced Salt Solution two times and then lysed in cold 95% ethanol for 20 min at room temperature. Cell lysate was collected and counted for radioactivity by the gamma-counter (Packard Instruments, Waltham, MA, USA). Radioiodide uptake not mediated by NIS was excluded by performing the assay in the presence of 3 mM perchlorate (ClO − 4), a competitive inhibitor of iodide uptake by NIS. Experiments were performed in triplicates.The uptake was normalized to the cellular protein concentration measured with Bio-Rad protein assay.

### Statistical analysis

Statistical analysis was performed by SPSS22.0. Data are presented as mean ± SEM. For comparison among two groups, Student’s *t* test was used. For multiple comparisons, ANOVA was used for initial analyses followed by Fisher’s protected least significant difference for post hoc analyses. Differences with *P* < 0.05 were determined as statistically significant.

## Results

### Effects of PLX4032 and PD98059 on NIS expression

**Fig. 1:**
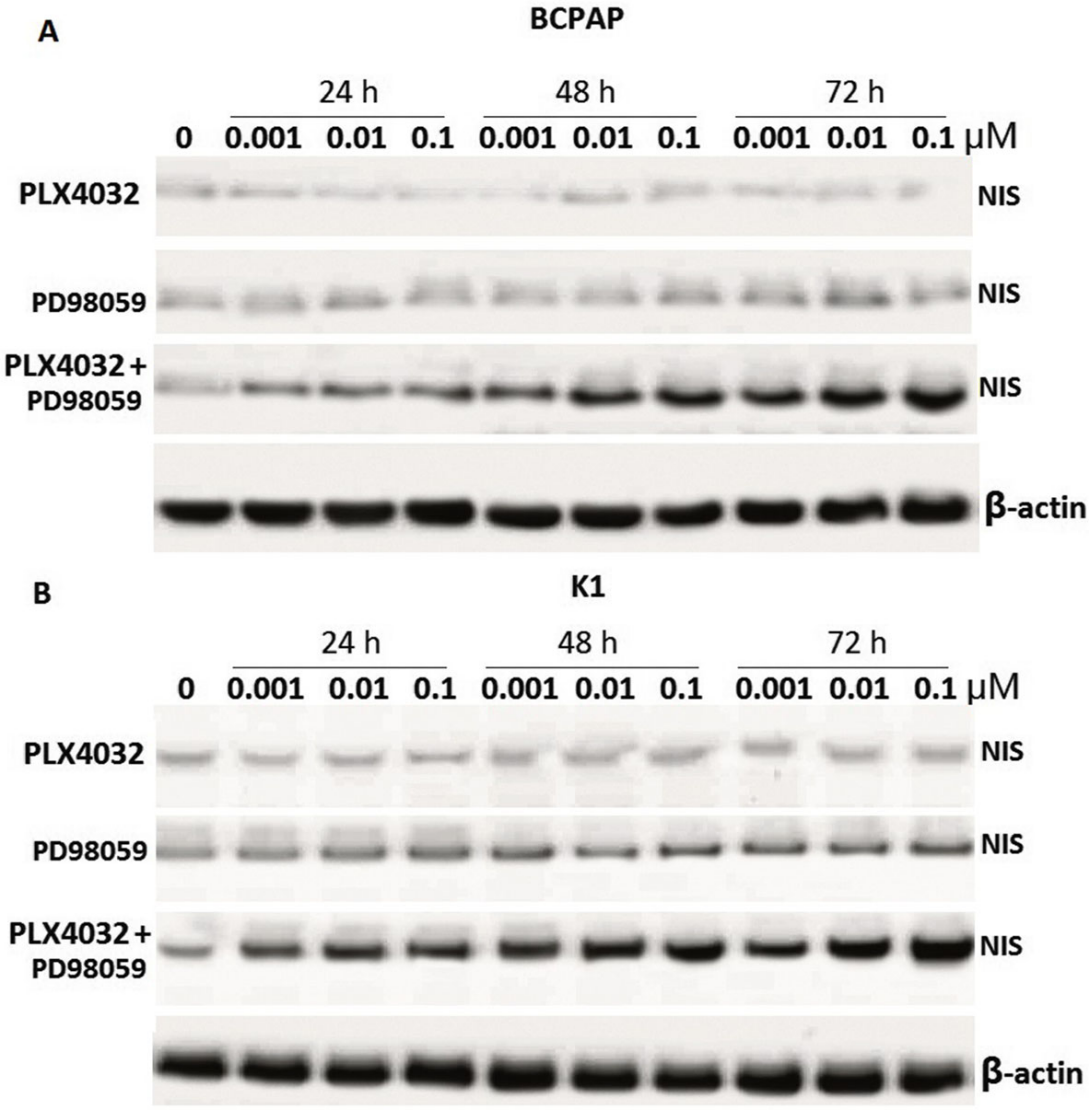
Western blot of NIS in lysates of BCPAP cells (**a**) and K1 cells (**b**) treated with PLX4032 (0.001–0.1 μM) or PD98059 (0.001–0.1 μM) or their combination for indicated times

BCPAP and K1 cells were exposed to increasing concentrations of PLX4032 (0.001 μM,0.01 μM, 0.1 μM) or PD98059 (0.001 μM, 0.01 μM, 0.1 μM) or with their association for 24 h, 48 h and 72 h. NIS protein was detected by western blot (Fig. 1a and b). The results showed that PLX4032 alone did not significantly increase NIS protein levels, and PD98059 alone slightly increased NIS protein levels, especially in K1 cells. However, combined treatment significantly increased NIS protein in a dose- and time- dependent way.

### Effects of PLX4032 and PD98059 on radioiodine uptake

**Fig. 2:**
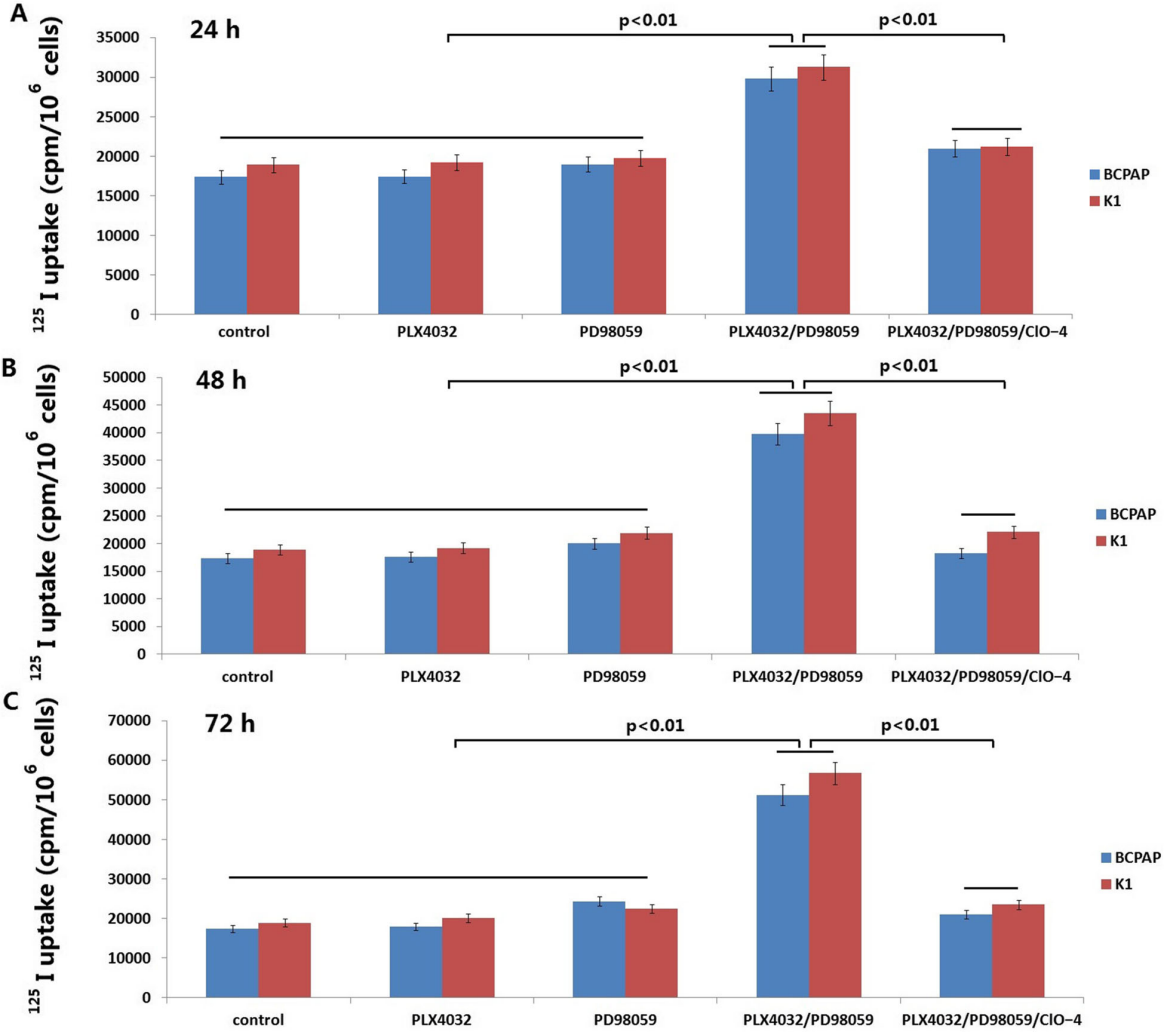
^125^I accumulation in BCPAP and K1 cells. BCPAP and K1 cells were treated with PLX4032 (0.001–0.1 μM) or PD98059 (0.001–0.1 μM) or their combination or/and NaClO4 for 24 hs (**a**), 48 hs (**b**) and 72 hs (**c**)

As shown in Fig. 2, radioiodine uptake was not significantly increased in the BCPAP and K1 cells with PLX4032 (0.001–0.1 μM) treatment for 24–72 h, but slightly increased in BCPAP and K1 cells with PD98059 (0.001–0.1 μM) treatment. However, combination of PLX4032 and PD98059 significantly increased radioiodine uptake in both of the two cell lines respectively, but suppressed iodide uptake by 3 mM perchlorate (ClO^−^ _4_), a competitive inhibitor of iodide uptake by NIS. It is suggested that Iodine uptake was specifically dependent on NIS because it was blocked by NaClO4 (Fig. 2).

### PLX4032 failed to increase NIS and NIS-mediated radioiodine uptake due to its activation of ERK signaling

**Fig. 3:**
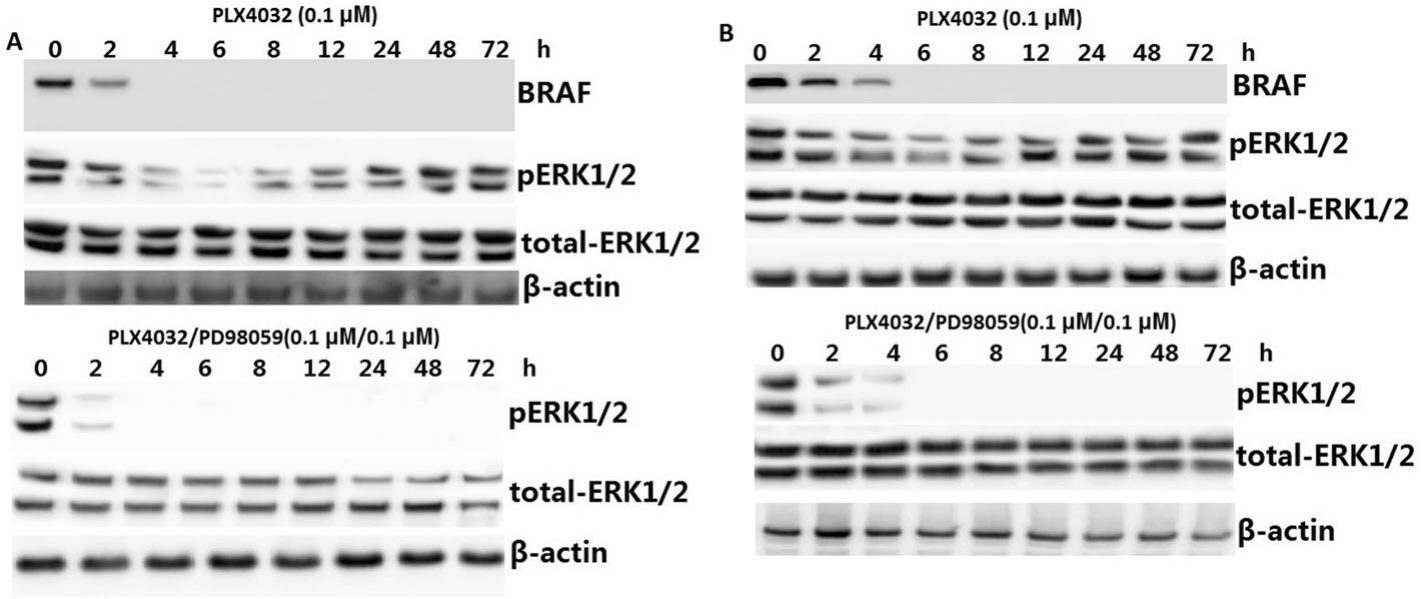
Western blot of BRAF and pERK levels in lysates of BCPAP cells (**a**) and K1 cells (**b**) treated with PLX4032 (0.1 μM) or PLX4032 (0.1 μM)/PD98059 (0.1 μM) for indicated times

PLX4032 (0.1 μM) treatment alone resulted in completely inhibition of BRAF in 6–8 hs by western blot assay (Fig. 3a) in BCPAP cells. But PLX4032 (0.1 μM) treatment resulted in a transient inhibition of pERK expression, but quickly recovery from ERK1/2 activation inhibition by PLX4032 treatment in 8 h, and gradually reached the high levels at 24 hs and matained this levels for 72 h (Fig. 3a). However, combined treatment of PLX4032 and PD98059 completely inhibited ERK1/2 activation in BCPAP cells (Fig. 3a). PLX4032 or PD98059 (0.1 μM) treatment has the same results on K1 cells (Fig. 3b).

## Discussion

Radioiodine ablation is the classical and standard treatments for thyroid cancer, which takes advantage of the unique iodide-transporting function of NIS in the thyroid cell membrane. However, the expression of iodide-metabolizing gene NIS is reduced in thyroid cancer, resulting in the reduction of iodide accumulation in the thyroid cells, particularly in dedifferentiated carcinoma. The decrease of NIS expression level directly leads to the reducion of iodine accumulation capacity in thyroid gland cells and the resistance to radioiodine therapy, leading to the treatment failure [20]. Therefore, understanding the mechanism of NIS and iodine treatment resistance is very important to overcome the radioiodine therapy resistance.

The BRAF^V600E^ mutation is the most common genetic change in thyroid cancer, particularly in papillary thyroid cancer (PTC) [4]. BRAF V600E mutation could abnormaly activate RAS-BRAF-MEK-MAP kinase (MAPK) pathway, leading to the thyroid tumorigenesis [19]. Several studies have demonstrated that B-RafV600E represses NIS expression [21, 22], but restoring NIS expression in thyroid cells when inhibiting the BRAFV600E/MEK pathway or silencing of BRAFV600E expression [7]. Clinical small sample size trials with MEK or BRAF inhibitors unfortunately failed to meet high expectations [23].

In our study, we demonstrated that inhibition of BRAF/MEK by PLX4032 alone did not significantly induce NIS expression and increase radioiodine uptake in both of the two cell lines, and PD98059 alone slightly increased NIS protein levels and radioiodine uptake in both of the two cell lines, especially in K1 cells. However, combination of PLX4032 and PD98059 significantly increased NIS protein levels and radioiodine uptake in both of the two cell lines, suggesting that inhibiting both BRAF/MEK and ERK1/2 can produce stronger redifferetiation effect.

Previous studies have found that PLX4032 treatment can cause a transient decrease of ERK1/2 activity in BRAFV600E-positive PTC cells, followed by the rebound of ERK1/2 activity in a time-dependent manne [17]. In our study, we found that PLX4032 (0.1 μM) treatment resulted in completely inhibition of pERK expression in 6–8 hs, but rebound from 8 h, and gradually reached the high levels at 24 hs and retained this levels for 72 h. However, PLX4032 combined with PD98059 completely prevented the reactivation of ERK1/2 in both of the cells. Our data shows that combination therapy by inhibiting the rebound of ERK1/2 activation induced NIS expression and increased radioiodine uptake in the two PTC cells.

## Conclusion

In conclusion, our study showed that PLX4032 combined with PD98059 can induce NIS expression and increase radioiodine uptake in PTC cells harboring *BRAF*^V600E^ though effectively preventing ERK1/2 activity rebound. Our study provides a theoretical basis for combining PLX4032/PD98059 and ^131^I treatment in in vivo expriments and clinical studies, which is also a promising treatment protocol for BRAF ^V600E^ positive PTC.

## Abbreviations

NIS: Sodium/iodide symporter
RAI: Radioiodide-refractory
MAPK: Mitogen-activated protein kinase
TSHR: Thyroid-stimulating hormone receptor.
